# PSMA-Specific CAR-Engineered T Cells for Prostate Cancer: CD28 Outperforms Combined CD28-4-1BB “Super-Stimulation”

**DOI:** 10.3389/fonc.2021.708073

**Published:** 2021-09-29

**Authors:** Gaia Zuccolotto, Alessandro Penna, Giulio Fracasso, Debora Carpanese, Isabella Monia Montagner, Silvia Dalla Santa, Antonio Rosato

**Affiliations:** ^1^ Department of Surgery, Oncology and Gastroenterology, University of Padua, Padua, Italy; ^2^ Department of Medicine, University of Verona, Verona, Italy; ^3^ Veneto Institute of Oncology IOV - IRCCS, Padua, Italy

**Keywords:** cancer immunotherapy, prostate cancer, CAR-T, PSMA, CAR generations

## Abstract

Prostate cancer (PCa) is the second leading cause of malignancy-related mortality in males in the Western world. Although treatment like prostatectomy and radiotherapy for localized cancer have good results, similar positive outcomes are not achieved in metastatic PCa. Consequently, these aggressive and metastatic forms of PCa urgently need new methods of treatment. We already described an efficient and specific second-generation (2G) Chimeric Antigen Receptor (CAR) against Prostate Specific Membrane Antigen (PSMA), a glycoprotein overexpressed in prostate cancer and also present on neovasculature of several tumor entities. In an attempt to improve efficacy and *in vivo* survival of anti-PSMA 2G CAR-T cells, we developed a third generation (3G) CAR containing two costimulatory elements, namely CD28 and 4-1BB co-signaling domains, in addition to CD3ζ. Differently from what described for other 3G receptors, our third generation CAR disclosed an antitumor activity *in vitro* similar to the related 2G CAR that comprises the CD28 co-signaling domain only. Moreover, the additional costimulatory domain produced detrimental effects, which could be attributed to an increased activation-induced cell death (AICD). Indeed, such “superstimulation” resulted in an exhausted phenotype of CAR-T cells, after prolonged *in vitro* restimulation, a higher frequency of cell death, and an impairment in yielding sufficient numbers of transgenic T lymphocytes. Thus, the optimal combination of costimulatory domains for CAR development should be assessed cautiously and evaluated case-by-case.

## Introduction

Adoptive cell therapy (ACT) with T lymphocytes expressing Chimeric Antigen Receptors (CAR) is at the forefront of current immunotherapeutic strategies. Successful results have been reported against leukemia (1–3), while solid tumors are still troublesome (4–6). One of the first objectives in CAR-based therapy is to identify valid targets to ensure patient safety and not only tumor rejection. In the context of Prostate Cancer (PCa), an interesting target is the Prostate Specific Membrane Antigen (PSMA), which is a well-described tumor-associated antigen. In particular, PSMA expression levels differentiate normal and cancerous prostatic tissues. Additionally, targeting PSMA could have an antiangiogenic effect since its expression has been observed on the neovasculature of several tumors. In this scenario, therefore, PSMA represents an ideal target; indeed, in many studies it is currently used not only for therapeutic strategies but also for imaging (7).

The choice of the target is certainly crucial to improve CAR-based therapies, but at the same time the efficiency of CAR T cells relies on an efficient expansion and persistence *in vivo* (8–10). Currently, it is not completely clear how CAR structure affects these characteristics. Indeed, CAR T cells have often shown potent *in vitro* cytolysis, but limited expansion, persistence, and tumor control in patients (11–14). It is increasingly evident that CAR structure has an important impact on T cell function, however much work remains to be carried out to delineate the relevance of all CAR moieties to optimize CAR constructs, and to assure enhanced antitumor potency, proliferative capacity, and persistence to transgenic T cells (15, 16). In this regard, T cells with a second-generation CAR (2G CAR) that allows CD28 co-stimulation present an enhanced antitumor function, while other stimulatory molecules could completely modify the effector functions of the transduced T cells (17). For example, it has been reported that in the third-generation CAR (3G CAR) the matched co-stimulation of CD28 with 4-1BB improves T-effector memory cell differentiation and protects cells from apoptosis (18). In the light of all these findings, learning more about the CAR structure and how its structure could affect the characteristics of T cells is essential.

With the final aim to optimize the efficacy of a CAR T cell therapy for PCa, we developed an anti- PSMA 3G CAR to enhance the biological properties of a 2G CAR construct already described (19). Notwithstanding, the 3G CAR containing the CD28 and 4-1BB costimulation molecules did not prove more effective than the 2G CAR with only one costimulatory domain (CD28). Moreover, the effect of additional signaling modules not only was not additive (20–24), but even detrimental. In particular, the addition of costimulatory domains increased activation induced cell death (AICD) in T cells expressing the 3G CAR, as a result of augmented FasL expression and induction of an exhausted status that appears with poor cytokine production, reduced expansion, an elevated percentage of apoptosis, and a significant expression of inhibitory receptors.

## Materials and Methods

### Cell Lines

The following human prostate carcinoma cell lines were used: LNCaP, PC3, and PC3-PSMA. PC3-PSMA, a PC3 derivative cell line stably expressing human PSMA, has been previously described (25). For virus production, we used the 293T (human embryonic kidney cell line) cell line obtained from the American Type Culture Collection (ATCC). Cells were cultured in RPMI 1640 (EuroClone, Milan, Italy) supplemented with 10% (v/v) heat-inactivated fetal bovine serum (Gibco BRL, Paisley, UK), 2 mL glutamine (Lonza, Verviers, Belgium), 10 mM HEPES (Lonza), 100 U/mL penicillin/streptomycin (Lonza), hereafter referred to as complete medium. Cell lines were maintained at 37°C in a humidified atmosphere containing 5% CO_2_. Firefly luciferase (fluc)-expressing PC3 and PC3-PSMA cell derivatives were obtained by viral transduction, as previously described (19).

### Vector Design

The transfer vector #945.pCCL.sin.cPPT.SV40ployA.eGFP.nCMV.hPGK.deltaLNGFR.Wpre is a self-inactivating (SIN) HIV-derived vector, which has been previously described (19) and carries a minCMVPGK divergent bidirectional promoter driving the simultaneous expression of two genes in antisense orientation. The transfer vectors used in this study contained the anti-hPSMA 2G or 3G CAR sequences under the control of the hPGK promoter, and the eGFP (enhanced Green Fluorescent Protein) under the control of minCMV. The synthetic genes containing the ScFvD2B-MycTag-CD28-CD3z (2G) or ScFvD2B-MycTag-CD28-4-1BB-CD3z (3G) chimeric sequences were custom synthetized by GeneArt, Life Technologies (Regensburg, Germany), and ligated into the #945 transfer vector by the restriction enzymes RsrII and SalI.

### T Cell Transduction

The anti-PSMA CAR/eGFP lentiviral transfer vector (LV) and viral particle production in 293 T cells have been previously described (19). We used PBMC from healthy donors to generate CAR-T cells. PBMC were activated for 48 hours (hrs) with OKT-3 (50 ng/mL; Ortho Biotech Inc) and human IL-2 (hIL-2, 300 U/mL; Proleukin; Novartis Pharmaceuticals). Then, T cells were transduced by LVs with a TU/ml infection of 05-5 x10^7^, as previously described (19). Briefly, the viral supernatant was added to T cells for 18 hours at 37°C and 5% CO_2_, with protamine sulfate (40 mg/mL; Sigma- Aldrich) and hIL-2 (500 U/mL). Fresh complete medium containing hIL-2 (100 U/mL) was then replaced to the viral supernatant. Seventy-two hrs later, we analyzed CAR and eGFP expression. Every week CAR-T cells were stimulated with irradiated (60 Gy) PC3-PSMA at a 10:1 ratio. Complete medium with fresh IL-2 was changed every 3 days.

### Phenotypic Analysis

All experiments were carried out on a flow cytometer FACSCalibur (BD Bioscience), and data obtained were analyzed using FlowJo software (TreeStar). CAR expression was evaluated through anti-c-myc mAb (clone 9E10; Sigma-Aldrich) or the relative isotype control (mouse IgG1, Southern Biotech, Milan, Italy), followed by a secondary antibody (PE-conjugated goat anti-mouse IgG; Southern Biotech). T cell phenotype was evaluated using mAb to CD62L, CCR7, CD27, CD28, CD57, PD-1, TIM-3, LAG-3 (all from eBioscience), Annexin V and FasL (from BD Bioscience), and the relative isotype controls purchased from the same companies. All FACS plots presenting CAR T cell phenotype data were conducted on gated CAR^+^ cells.

### Cytotoxicity Assay

As previously reported (19), the cytotoxic activity of 2G and 3G CAR T cells was assessed in a 4 h ^51^Cr-release assay. Target cells were PC3-PSMA, LNCaP, and PC3. Briefly, effector cells were incubated with 2x10^3 51^Cr-labeled targets at various E/T ratios in triplicate wells on 96-well round-bottom plates. The percentage of specific lysis was calculated as previously described (19).

### Cytokine Release Assay

IFN-γ, IL-2, and TNF-α production were evaluated using specific ELISA kits (Thermo Scientific), according to the manufacturer’s instructions. Briefly, 1x10^6^ CAR T cells were seeded for 12 hrs with 1x10^6^ target cells (PC3 or PC3-PSMA), E/T ratio 1:1, in triplicate wells on 96-well round bottom plates. Negative and positive controls were represented by CAR T cells that remained unstimulated (medium only) or treated with 40 ng/mL of PMA and 4 mg/mL of ionomycin (Sigma-Aldrich). Cytokine secretion in supernatants was then measured on a VICTOR X4 (Perkinelmer).

### Statistics

A Student’s t test was used to compare two value sets, while we used one-way ANOVA when three groups were involved. Histograms represent mean values ± standard deviations. P < 0.05, P < 0.01, or P < 0.001 were indicated by *, ** or ***, respectively. GraphPad Prism 7.0 software was used for all the statistical analyses.

## Results

### Construction of a Second- and Third-Generation Anti-PSMA CAR

**Figure 1:**
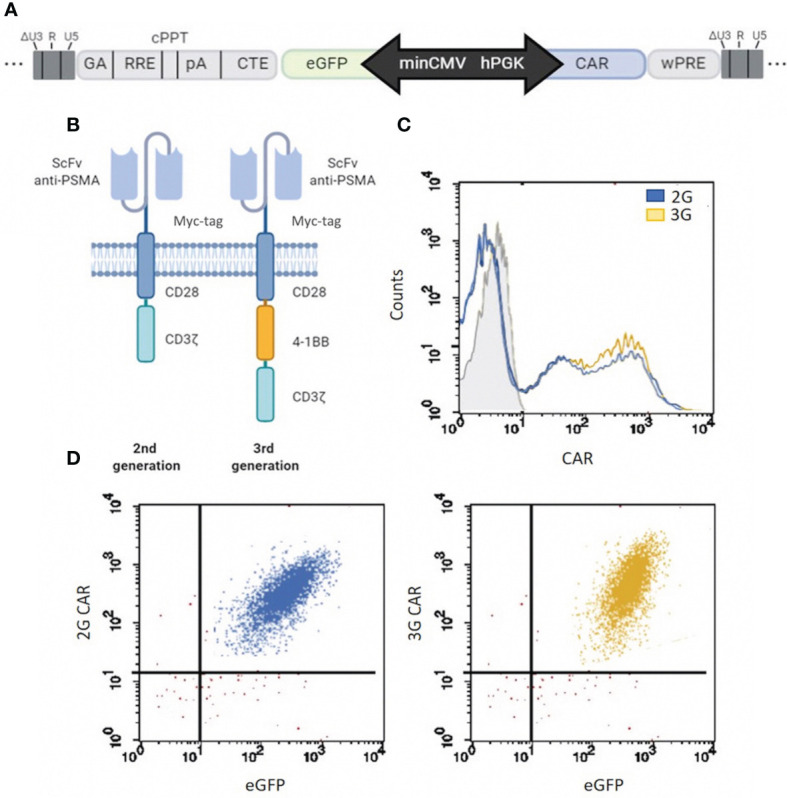
CAR structure development and expression. **(A)** Map of the minCMVPGK bidirectional promoter. CAR gene expression is regulated by the hPGK promoter, while the reporter gene (eGFP) is under the control of the minCMV promoter. **(B)** Schematic representation of the two different anti-PSMA CAR designs. 2G and 3G are second- and third-generation CARs, respectively, whose antigen-binding domain comes from antibody IgGD2B (26), and contain a myc tag for cytofluorimetric detection. The two CARs are different for the intracellular costimulatory domains, which are composed of a CD28 plus CD3ζ moieties in 2G or CD28, 4-1BB (CD137) and CD3ζ elements in 3G. **(C)** 2G and 3G CAR transduction. Flow cytometry of CAR expression (2G in light blue and 3G in yellow) in T cells at 4 weeks post LV transduction. Grey histogram represents the isotype control. **(D)** Co-expression of CAR and eGFP in 2G and 3G populations of LV-transduced T cells. More than 90% of cells co-expressed both CAR (c-myc) and the reporter gene (eGFP). The events of the dot plot were gated on total viable cells.

The 2G and 3G CAR sequences were inserted into a Lentiviral Vector (LV) carrying a minCMVPGK bidirectional promoter (19) that allows the simultaneous and coordinated expression of two genes, a reporter gene (eGFP) and the anti-PSMA CAR (**Figure 1A**). The 2G and 3G CAR sequences were designed that encoded the following components: the single-chain variable fragment (scFv) of the anti-PSMA antibody IgGD2B (26), a myc tag for cytofluorimetric detection, and the CD28 co-stimulatory molecule linked directly to the CD3ζ sequence, for the 2G CAR; the 3G CAR presented a second co-stimulatory molecule (4-1BB) within the CD28 and CD3ζ domains (**Figure 1B**). The anti-PSMA scFv (scFvD2B) used for the development of CAR structures is well described (25), and presents very promising characteristics, especially the high affinity for the target. T cells were transduced with LV 2G and 3G CAR PSMA/eGFP. One month after transduction, more than 90% of cells were CAR^+^ (**Figure 1C**), with a balanced expression of both the CAR (2G and 3G) and the reporter gene (eGFP) sustained by the bidirectional LV (**Figure 1D**).

### 2G and 3G CAR T Cells Exhibit a Similar Trend in the Accumulation of the CAR-Expressing Subset, but the 3G Population Rapidly Undergoes a More Differentiated Effector Memory Phenotype

**Figure 2:**
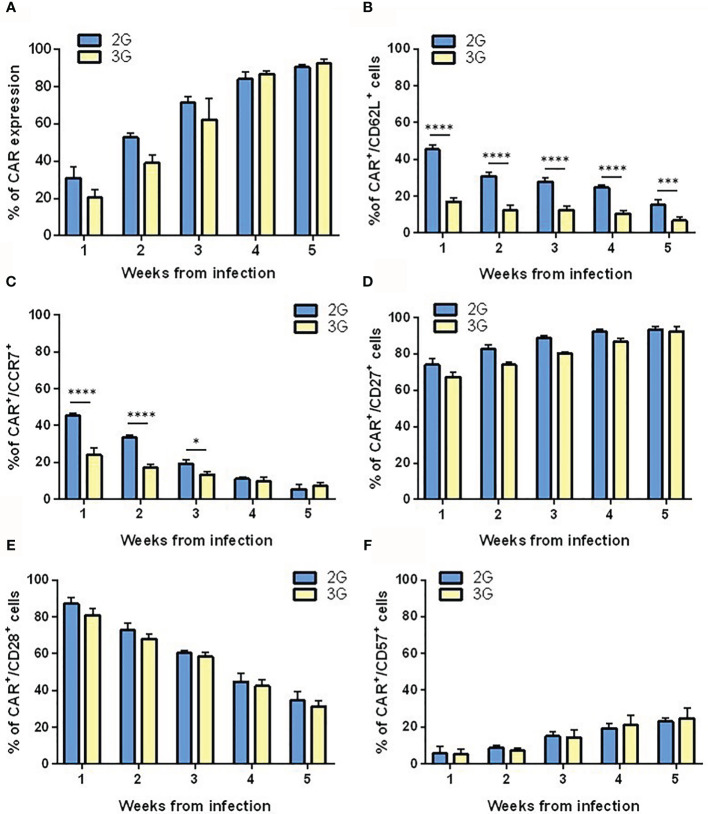
Differentiation pattern of the 2G and 3G CAR-expressing populations. **(A)** 2G and 3G CAR expression after stimulation. Histograms report the percentage of 2G (light blue) and 3G (yellow) CAR^+^ cells in LV CAR hPSMA/eGFP transduced T cell populations at different weeks from infection. Expression of **(B)** CD62L, **(C)** CCR7, **(D)** CD27, **(E)** CD28, and **(F)** CD57. All surface markers were evaluated in 2G and 3G CAR-expressing populations at different weeks from infection, as assessed by flow cytometry. Figures show the mean +/- SD of at least three independent experiments. *p < 0.05, ***p < 0.001, ****p < 0.0001 by two-way ANOVA.

To generate T cells expressing 2G or 3G CAR anti-PSMA, we used a previously described (19) expansion protocol that involved weekly restimulations with PC3-PSMA cells. Thus, both CAR T cell populations had the chance to encounter the antigen, which sustained the expansion of the CAR-expressing subset (**Figure 2A**). To characterize the state of differentiation of CAR-transduced T lymphocytes in the post-infection period and during antigenic restimulation, we cytometrically analyzed the expression of different surface markers, namely CD62L, CD27, CD28, CCR7, and CD57. One week after transduction, 2G CAR T cells presented the typical characteristics of early effector T cells, as shown by the high expression of CD62L (**Figure 2B**), the presence of CCR7 (**Figure 2C**), CD27 (**Figure 2D**) and CD28 (**Figure 2E**), and the low expression of CD57 (**Figure 2F**). Conversely, 3G CAR T cells showed a significant difference in the expression of CD62L and CCR7 already at the first week post-transduction, and very rapidly acquired a more differentiated effector memory phenotype. Following re-stimulation with the antigen, both T cell populations down-modulated CD62L, CCR7, CD28 and presented a slight increase in CD57 expression, thus progressively acquiring an intermediate effector memory phenotype. Moreover, we observed that T cells are mostly stem cell memory (TSCM) 3 days after transduction, to progressively switch into T central memory (TCM) and T effector memory (EM) after 15 and 25 days, respectively (**Supplementary Figure 1**).

### 2G and 3G CAR T Cells Show Comparable Tumor Cell Killing and Cytokine Production Following Exposure to PSMA^+^ Cells

**Figure 3:**
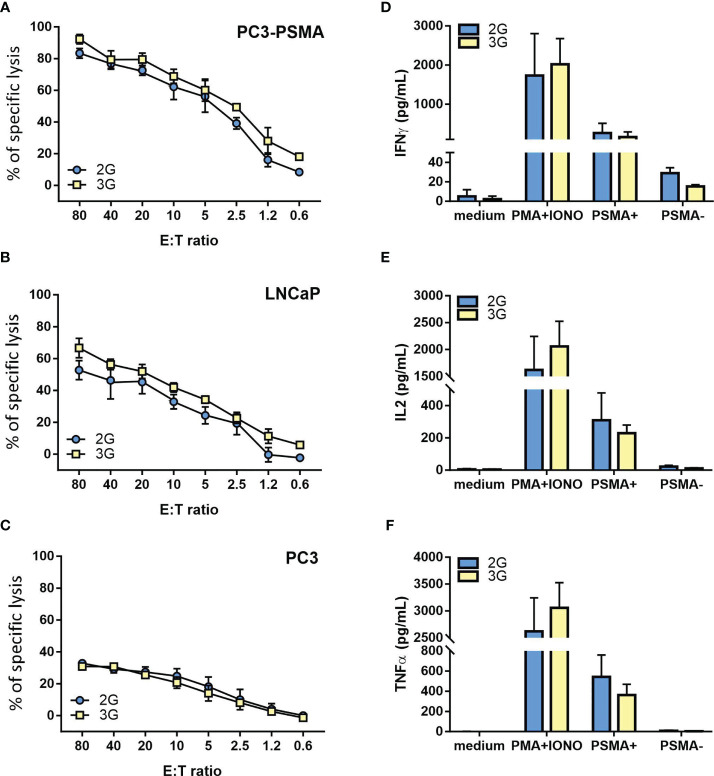
Functional characterization of the 2G and 3G CAR-expressing populations. **(A–C)** Lytic activity of the 2G (light blue) and 3G (yellow)-CAR expressing populations. Cytotoxicity was analyzed at day 15 post-transduction; as target cells, **(A)** PC3-PSMA, **(B)** LNCaP, and **(C)** PC3 were used. **(D–F)** Cytokine release upon antigen stimulation. **(D)** IFN-γ, **(E)** IL-2 and **(F)** TNF-α. Cytokine release was evaluated 15 days after T cell infection by stimulating 2G and 3G CAR populations with PC3-PSMA or PC3 cancer cell lines. Negative and positive controls were represented by 2G and 3G CAR T cells treated or not with PMA/Ionomycin. Figures show the mean +/- SD of 3 independent experiments.

Both the 2G and 3G CAR populations lysed the PSMA-transfected PC3 cells at high levels (**Figure 3A**). Moreover, they efficiently recognized LNCaP cells, a target that naturally harbors the PSMA antigen (**Figure 3B**), while sparing the antigen-negative counterpart (**Figure 3C**). Other than exerting a relevant cytotoxic activity, both generations of CAR-transduced T cells also produced high and comparable levels of IFN-γ, IL-2, and TNF-α in response to PSMA-expressing tumor targets, but not against PSMA negative control cells (**Figures 3D–F**).

### 3G CAR T Cells Become Exhausted During *In Vitro* Expansion

**Figure 4:**
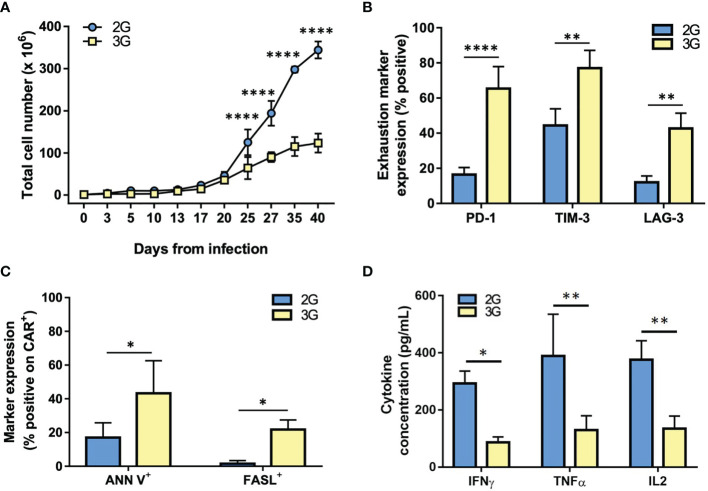
3G CAR T cells undergo exhaustion during *in vitro* expansion. **(A)** Cell expansion of the 2G (light blue) and 3G (yellow) CAR-expressing populations upon weekly antigen stimulation. **(B)** Quantification of exhaustion marker expression at 25-35 days from transduction. **(C)** Quantification of ANN V^+^ and FASL^+^ in 2G and 3G CAR populations at 25-35 days from transduction. **(D)** Cytokines production upon stimulation with PC3-PSMA target cells at 25-35 days from transduction. Figures show the mean +/- SD of 3 independent experiments. *p < 0.05, **p < 0.01, ***p < 0.001****p < 0.0001 by two-way ANOVA.

During *in vitro* culture and restimulation, 2G CAR T subset progressively expanded and accumulated up to 6 weeks, the last time point tested. Conversely, 3G CAR T cells strongly reduced their proliferation by week 4 from transduction, with a significant difference in the total CAR T cell yield from day 25 to day 40 (p<0.000; **Figure 4A**). By day 25, furthermore, CD28-ζ-4-1BB (3G) CAR T cells showed a cell surface profile consistent with exhaustion, including a higher and significant difference in the expression of PD-1 (p<0.0001), TIM-3 (p=0.0019) and LAG-3 (p=0.0031), as compared to the 2G CAR T cell population (**Figure 4B**). Moreover, 3G CAR T cells showed higher rates of apoptosis (Ann V^+^; p<0.0031) in comparison to the 2G CAR T cell population, likely associated to the induction of the FAS-FASL pathway (FASL^+^; p=0.0454; **Figure 4C**). In addition, at these late stages of culture 3G CAR T cells produced lower levels of IL-2, TNF-α, and IFN-γ following exposure to PSMA^+^ cells, as compared with 2G CAR T cells (**Figure 4D**). Together, these phenotypic and functional data demonstrate that 3G CAR T cells become rapidly exhausted and have a limited expansion during *in vitro* culture, whereas similar effects do not occur in 2G CAR T cells stimulated in the same manner.

## Discussion

Remarkable clinical outcomes were evidenced in hematological malignancies after injection of CD19 CAR T cells, overall establishing the concept that CAR therapies represent one of the most effective immune-based treatments of cancer (27, 28). Differently, solid tumors are still an open battlefield for immunotherapeutic strategies, with unsatisfactory results obtained thus far even only for the intrinsic difficulties to the treatment like the choice and the safety of the target antigens.

Within solid tumors, PCa is the most frequent male tumor in Europe and the US (29) and expresses some typical surface antigens that can be targeted by ACT. Among these, PSMA is a well-known tumor-associated antigen that is overexpressed in PCa, and against which we have already reported the development of a 2G CAR (19) based on a high affinity specific mAb (26). Despite an overall good activity, *in vivo* persistence of related CAR T cells was however reduced. In this regard, several trials have demonstrated the importance to augment costimulation and reduce exhaustion of T cells, to improve the persistence and efficacy of CAR T cells (30).

On the other hand, it is still partly unclear how the different potencies of the chimeric receptors depend on their structure. To this purpose, here we developed a third generation CAR that harbors the same high affinity antigen recognition domain but with different serial endodomains combining CD28 and 4-1BB costimulatory elements with CD3ζ. Thereafter, 2G and 3G CARs were assessed and compared for functional activity in cultures of human primary T lymphocytes. Both CARs proved efficient to respond specifically to only PSMA^+^ tumor cell lines *in vitro*. However, starting already from just the first week post-transduction, the 3G CAR T cell population underwent the acquisition of a more differentiated effector phenotype that appeared more prone to AICD, as compared to the 2G counterpart. Moreover, while the expansion rate of either CAR T cell populations was comparable during the first three weeks of culture, thereafter 2G CAR T cells continued to expand whereas 3G CAR-transgenic lymphocytes reached a plateau and then started to die. Thus, the 3G CAR produced a “super stimulation” that accelerated apoptosis and exhaustion of the transgenic population during *in vitro* expansion. This exhaustion was characterized by a diminished cytokine production, a poor proliferative capacity, and a high expression of exhaustion markers. Likely, the excess of co-stimulatory signals led to an increased FasL expression that in turn induced AICD, and ultimately precluded the generation of sufficient numbers of 3G CAR to proceed with *in vivo* testing. Anyway, this expansion protocol was developed to recapitulate the *in vivo* antigen exposure, through the use of PSMA^+^ cell lines as periodic stimulation. However, it is possible that 3G CAR T cells could perform better adapting the settings of culture conditions to those used in the clinical procedure, addressing the differences in the exhaustion phenotype and to reach a sufficient number of cells to perform *in vivo* experiments.

Overall, our results underline the importance of optimizing CAR construction, and highlight the relevance to understand how receptor structure acts on transgenic T cell functionality, especially now that it is clear that some CARs cause exhaustion and impaired T cell activity. For the treatment of solid tumors, in particular, the role of T-cell exhaustion is crucial to assure the persistence of the adoptively transferred cells (30–32). In this regard, despite some authors assert that third-generation CAR-T cells work better than their equivalent second-generation CAR other groups instead assume that 3G-CAR, containing both CD28 and 4-1BB, is inferior to its own 2G counterpart containing only CD28 (20, 23, 33–36). Intriguingly and similar to what we observed, Hombach et al. reported that cytokine-induced killer (CIK) cells transduced with a second-generation CAR (CD28-CD3ζ) outperformed their relative third generation (CD28-OX40-CD3ζ) CAR-transduced counterparts, likely because the combined costimulation accelerated the maturation of CIK cells and made them more prone to apoptosis (18). Additionally, there is an increasing amount of evidence supporting the concept that extensive CAR signaling can be detrimental for T cell functionality (20, 23, 33–38). Thus, further research is needed to increase our understanding of how multiple costimulatory domains interact, in order to optimize CAR signaling and to attain a complete synergy from combinations.

Indeed, each costimulatory domain has unique properties, and it is unlikely that a single costimulatory domain will serve all purposes. Differences in the affinity of the scFv, the intensity of antigen expression, the probability of off-tumor toxicity, or the disease to be treated may influence the selection of the intracellular domain to enclose in the CAR structure. Therefore, 4-1BB stimulation of T cells may not be universally beneficial; rather, the overall outcome of 4-1BB signaling may depend on the scFv used and the presence of other costimulatory molecules.

In our 3G CAR, the presence of a high affinity scFv (26) and two signaling molecules result in overactivation of transduced T cells after antigen engagement, leading to AICD and exhaustion. Even though some preclinical studies have shown a superior functionality of 3G CAR T cells, our data are in line with other studies that show how 3G CAR T cells performed worse than their 2G counterparts (20, 35, 36). While the precise reasons of this discrepancy are still unclear, our findings support the concept that a combined costimulation better supports low affinity CAR T cells. Indeed, some works have shown how low affinity scFv combined to an increased costimulation, induce in CAR-T cells a slow differentiation, less exhaustion, and better proliferative capacity *in vitro* (20, 37, 38). Moreover, another key aspect in CAR design and development that could influence CAR functionality, is the proximity of the respective domains to the cell membrane (24, 39). Indeed, some studies have highlighted that expression of 4-1BB into CAR endodomain had detrimental effects, while expressing 4-1BB ligand (4-1BBL) on the surface enhanced efficacy (15, 37).

On the whole, it is increasingly evident that CAR functionality relies on an intricate interplay between scFv affinity, and number and position of the signaling domains. Thus, our and other works entail that the ideal CAR structure should be determined for every target and for each scFv, because an excessive stimulation could be detrimental. Moreover, these findings indicate that there is the necessity of increasing our knowledge about how molecular signaling may affects CARs, to help in choosing the right intracellular domain or combination of intracellular domains for each condition.

## Data Availability Statement

The original contributions presented in the study are included in the article/**Supplementary Material**. Further inquiries can be directed to the corresponding author.

## Author Contributions

GZ, IMM, and AP executed experiments. DC and SD analyzed the data. GZ and AR designed and supervised the study. GF contributed reagents/materials/analysis tools. GZ and AR wrote the manuscript. All authors contributed to the article and approved the submitted version.

## Funding

The research leading to these results has received funding from Fondazione AIRC under IG 2018–ID. 21354 project – P.I. AR; 5 per Mille 2019 - ID. 22759 program - G.L. AR, and the Ministry of Health-Alliance Against Cancer (MoH-ACC) project “Research project on CAR T cells for hematological malignancies and solid tumors” to AR.

## Conflict of Interest

The authors declare that the research was conducted in the absence of any commercial or financial relationships that could be construed as a potential conflict of interest.

## Publisher’s Note

All claims expressed in this article are solely those of the authors and do not necessarily represent those of their affiliated organizations, or those of the publisher, the editors and the reviewers. Any product that may be evaluated in this article, or claim that may be made by its manufacturer, is not guaranteed or endorsed by the publisher.

## Supplementary Material

Supplementary Figure 1 Phenotype of cultured 2G and 3G CAR T subsets in response to PSMA stimulation. **(A)** Quantification of CD45RA and CCR7 expression in 2G and 3G CAR T cells at 3, 14 and 25 days from transduction. T cell-derived subsets are: T_SCM_ (CD45RA^+^/CCR7^+^), T_CM_ (CD45RA^-^/CCR7^+^), T_EM_ (CD45RA^-^/CCR7^+/-^).

## Abbreviations

^51^Cr, chromium-51; 2G-CAR, second-generation CAR; 3G-CAR, third-generation CAR; ACT, adoptive cancer immunotherapy; AICD, activation-induced cell death; ATCC, American Type Culture Collection; Alpha MEM, minimum essential medium eagle, alpha modification; CAR-T, chimeric antigen receptor T; DMEM, Dulbecco’s Modified Eagle Medium; E/T, Effector/Target; eGFP, enhanced green fluorescent protein; FBS, Fetal Bovine Serum; IFN-γ, interferon-γ; ICD, intracellular costimulatory domain; LV, lentiviral transfer vector; mAb, monoclonal antibody;PBS, phosphate buffered saline; PCa, prostate cancer; PMA, phorbol-myristate-acetate; PSMA, human prostate-specific membrane antigen; RPMI, Roswell Park Memorial Institute; ScFv, single-chain variable fragment; SD, standard deviation; TME, tumor microenvironment; TMD, transmembrane domain.
