# Supplementary figures and images for: PSMA-Specific CAR-Engineered T Cells for Prostate Cancer: CD28 Outperforms Combined CD28-4-1BB “Super-Stimulation”
Source: Front Oncol. 2021 Sep 29;11:708073. doi: 10.3389/fonc.2021.708073 (PMC8511814; doi:10.3389/fonc.2021.708073)

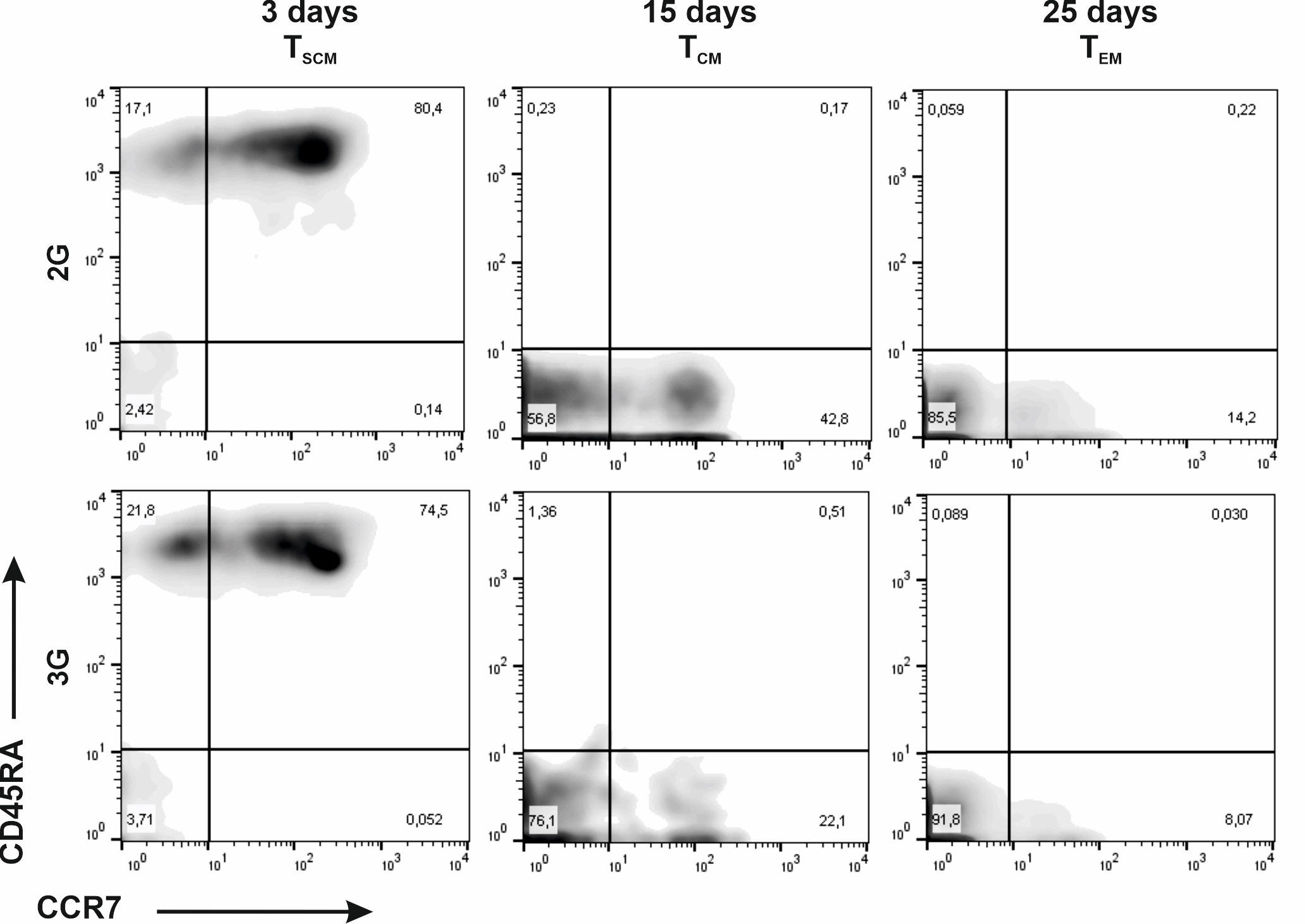

Supplement: Supplementary Figure 1 — Phenotype of cultured 2G and 3G CAR T subsets in response to PSMA stimulation. (A) Quantification of CD45RA and CCR7 expression in 2G and 3G CAR T cells at 3, 14 and 25 days from transduction. T cell-derived subsets are: TSCM (CD45RA+/CCR7+), TCM (CD45RA-/CCR7+), TEM (CD45RA-/CCR7+/-). [file Image_1.jpeg]
